# Case Report: Personalized diagnosis and treatment strategies for three cases of cancer of unknown primary based on molecular testing techniques

**DOI:** 10.3389/fonc.2025.1505271

**Published:** 2025-03-28

**Authors:** Yao Ding, Kexue Zhou, Kaiwen Fu, Xingyun Liao, Shuanglong Xiong, Chengxiang Yang, Mingyang Hu, Guanzhong Liang, Xianghua Zeng, Yongsheng Li, Donglin Wang, Yan Li

**Affiliations:** ^1^ Phase I Clinical Trial Ward, Chongqing University Cancer Hospital, Chongqing, China; ^2^ Department of Medical Oncology, Chongqing University Cancer Hospital, Chongqing, China; ^3^ Department of Pathology, Chongqing University Cancer Hospital, Chongqing, China

**Keywords:** cancer of unknown primary, gene expression profiling, tumor of origin, molecular testing techniques, personalized treatment

## Abstract

Cancer of unknown primary (CUP) is a malignancy characterized by metastatic disease at diagnosis with an unidentified primary site, accounting for 3–5% of all cancers. Despite significant advancements in cancer diagnosis and treatment in recent years, CUP management has been challenging due to its complexity and heterogeneity; therefore, its prognosis remains poor. This report presents three cases of CUP. The first case involved a 59-year-old female whose abdominal metastatic cancer was identified to be originating from a primary cervical cancer using a 90-gene panel; the disease was controlled with targeted immunotherapy. The second case was a 56-year-old male with cervical lymph node metastatic cancer; genetic testing suggested renal cancer as the primary site, and dual-targeted therapy resulted in approximately 28% tumor reduction. The third case involved a 71-year-old female with subcutaneous metastatic cancer, which was confirmed by genetic profiling to be related to breast cancer; she achieved stable disease after chemotherapy. Diagnosis and treatment of these three CUP cases demonstrated that molecular testing could significantly improve treatment outcomes and extend patient survival. Precision medicine based on molecular detection has shown substantial value in identifying the primary site of CUP, developing personalized treatment plans, and managing the disease. However, treatment costs and patient compliance remain challenging, necessitating further research to optimize both diagnostic and therapeutic strategies.

## 1 Introduction

Cancer of unknown primary (CUP) is a type of metastatic malignant tumor characterized by the presence of metastases at diagnosis without an identifiable primary site despite thorough investigation. CUP constitutes approximately 3–5% of all malignant tumors, although recent studies suggested a decline in their incidence to less than 2%. Due to challenges in diagnosis and treatment, the prognosis for CUP remains poor, with a median survival of 3 months and a one-year survival rate of less than 20% (1–3).

The etiology of CUP remains unclear; however, it is believed to involve a combination of factors, including genetic predisposition, environmental influences, and lifestyle choices. Pathological studies have suggested that CUP cells acquire significant invasiveness and metastatic potential at an early stage, thereby allowing metastases to form by the time of detection. The primary tumor may be too small or regressive, making it difficult to detect (4, 5). Advances in genomics and molecular biology have uncovered a range of genetic mutations and signaling pathway abnormalities associated with CUP, offering new insights into its pathogenesis (6). Treatment strategies for CUP often rely on empirical therapies, such as systemic chemotherapy, targeted therapy, and immunotherapy. The heterogeneous nature of CUP requires that each patient’s treatment plan be individualized, with the choice of therapy depending on the patient’s specific condition, the tumor’s histological type, and its molecular characteristics (7–9).

In recent years, molecular diagnostic techniques, particularly RNA sequencing, have increasingly been applied in the diagnosis of CUP. RNA sequencing analyzes the RNA expression profiles in tumor samples, offering critical information on gene expression that facilitates the identification of potential primary tumor sites. Studies have shown that RNA sequencing significantly improves the detection of the primary sites in CUP cases. By guiding treatments based on tumor types, this technique enhances patient outcomes and provides a solid foundation for developing personalized treatment strategies (10–14).

In this case series, we used a gene detection method based on the tumor tissue origin (90-gene panel, provided by Canhelp Genomics Co., Ltd., Hangzhou, China). This method is based on a pan-cancer transcriptome database containing 5434 specimens spanning 21 tumor types, constructed to maximize diagnostic accuracy. Gene selection employed the Support Vector Machine Recursive Feature Elimination (SVM-RFE) algorithm to identify a non-redundant set of 90 genes, ensuring optimal representation of tumor-specific expression patterns. The assay operates through a support vector machine (SVM) linear model that generates similarity scores (ranging from 0–100) for each tumor type based on the gene expression profile. These scores represent the likelihood of the tested sample matching the reference gene expression signature of each tumor type, with the highest score suggesting the most probable primary tumor origin. In this study, total RNA was isolated from biopsy samples, and cDNA synthesis was performed. Quantitative RT-PCR, based on the TaqMan detection system, was conducted using the ABI 7500 or MacroStone 96S platforms. Gene expression similarity scores were calculated using the classifier’s proprietary software. The results were interpreted in conjunction with clinical and pathological data to refine the final diagnosis (15). This approach effectively identified the primary tumor site, offering clinicians vital diagnostic information that guided personalized treatments. Tumor response evaluations for each case were conducted according to the RECIST 1.1 criteria for solid tumors (16).

## 2 Case report

### 2.1 Case 1

**Figure 1:**
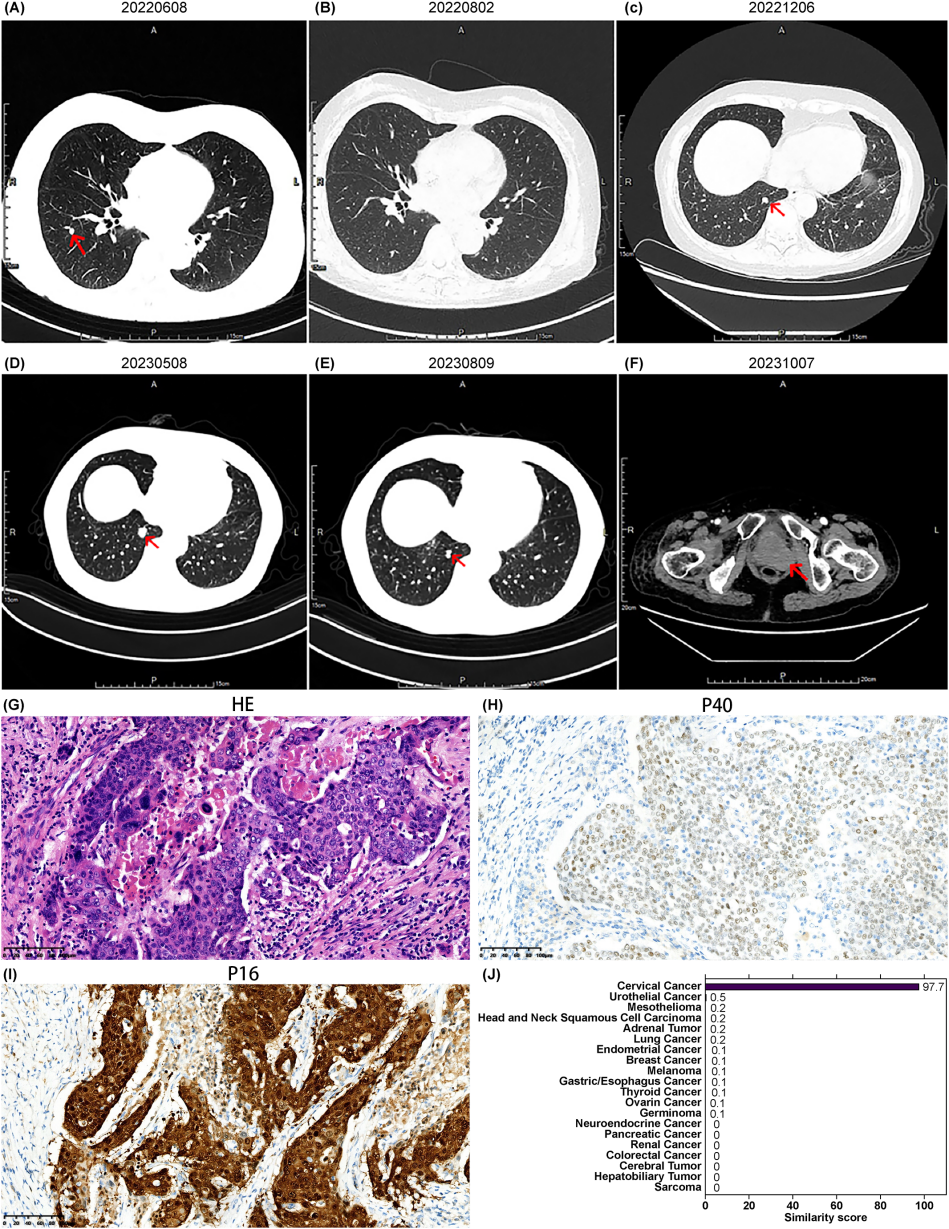
Imaging, pathology, and gene similarity score results of Case 1. **(A)** Newly detected right lung metastasis after four cycles of TP regimen chemotherapy. **(B)** Shrinkage of the right lung metastatic lesion on follow-up chest CT after two cycles of GP regimen chemotherapy. **(C)** New right lung lesions detected on chest CT on December 6, 2022. **(D)** Enlargement of the right lung lesion on chest CT on May 8, 2023. **(E)** Reduction of the right lung lesion observed on chest CT on August 9, 2023, after several cycles of targeted therapy based on the 90-gene test results. **(F)** New pelvic lesion detected on October 7, 2023, indicating disease progression. **(G)** Hematoxylin and eosin HE-stained pathological image of the retroperitoneal lesion, and immunohistochemistry results for **(H)** P40 and **(I)** P16 staining (20× magnification). **(J)** Gene similarity scores from the 90-gene test.

A 59-year-old Asian female presented with a pelvic mass detected during a health examination 4 days prior. She had no significant medical, family, or psychological history and had not undergone any previous genetic testing. Physical examination revealed no abnormal findings. The patient underwent total laparoscopic hysterectomy with bilateral salpingo-oophorectomy, left sacral ligament mass resection, pelvic adhesion separation, and pelvic drainage under general anesthesia on January 17, 2022. Postoperative pathology revealed malignant tumors with necrosis in the right and left retroperitoneal masses, consistent with metastatic adenocarcinoma. A PET-CT scan on January 29, 2022, did not detect any tumor lesions. On February 15, 2022, a pathological consultation at the First Affiliated Hospital of Chongqing Medical University confirmed squamous cell carcinoma based on the morphology and immunohistochemistry results. On March 8, 2022, a pathological consultation for complex cases at Chongqing University Cancer Hospital suggested a diagnosis of poorly differentiated carcinoma in the right retroperitoneum. Based on morphology and immunohistochemistry findings, the diagnosis was consistent with squamous cell carcinoma. Clinical correlation was recommended to rule out metastasis. Immunohistochemical results were as follows: Ki-67 (+, 30%), p63 (+), P40 (+), p16 (diffuse +), Syn (-), CK7 (partial +), CK19 (+), CEA (partial +), CDX2 (-), CK5/6 (+), ER(+), TTF-1(-), GATA-3 (+), and PAX-8 (-) (**Figures 1G–I**). The diagnosis was cancer of CUP. Following the National Comprehensive Cancer Network (NCCN) guidelines, the patient was treated with platinum-based chemotherapy. She subsequently received four cycles of the TP regimen (paclitaxel 270 mg on day 1 + cisplatin 35 mg on days 1–3, every three weeks), which she tolerated well. In June 2022, owing to disease progression (newly detected right lung metastasis; **Figure 1A**), the treatment was switched to the GP regimen (gemcitabine 1500 mg on days 1 and 8 + nedaplatin 120 mg on day 1, every three weeks) for two cycles, which the patient tolerated well. On August 2, 2022, re-evaluation showed stable disease with tumor reduction 25% (**Figure 1B**); however, the patient temporarily refused further chemotherapy and opted for follow-up observation.

In December 2022, re-evaluation revealed new bone metastases in the right iliac bone and ischium, with an increased size and number of lung nodules, indicating disease progression (**Figure 1C**). The 90-gene panel revealed a similarity score of 97.7 for cervical cancer as the tumor origin (**Figure 1J**), with a tumor mutation burden of 33.69 Muts/Mb. Following the NCCN and Chinese Society of Clinical Oncology (CSCO) guidelines for cervical cancer, palliative radiotherapy was administered to the right iliac bone and ischial metastases, along with two cycles of bevacizumab combined with paclitaxel and carboplatin, on December 14, 2022. In February 2023, the patient continued with a further four cycles of bevacizumab, paclitaxel, and carboplatin, with a post-treatment evaluation indicating stable disease. Minor enlargement of the lung lesions was subsequently observed (**Figure 1D**). Pembrolizumab immunotherapy was initiated in May 2023. During this period, disease stability was maintained, with a further reduction in lung nodules (**Figure 1E**). However, a follow-up CT scan on October 7, 2023, revealed disease progression with a new vaginal stump mass (**Figure 1F**). The progression-free survival (PFS) time after gene-directed treatment was 9 months, which was significantly better than that after frontline therapy. The patient refused further treatment and died in January 2024 owing to disease progression.

### 2.2 Case 2

**Figure 2:**
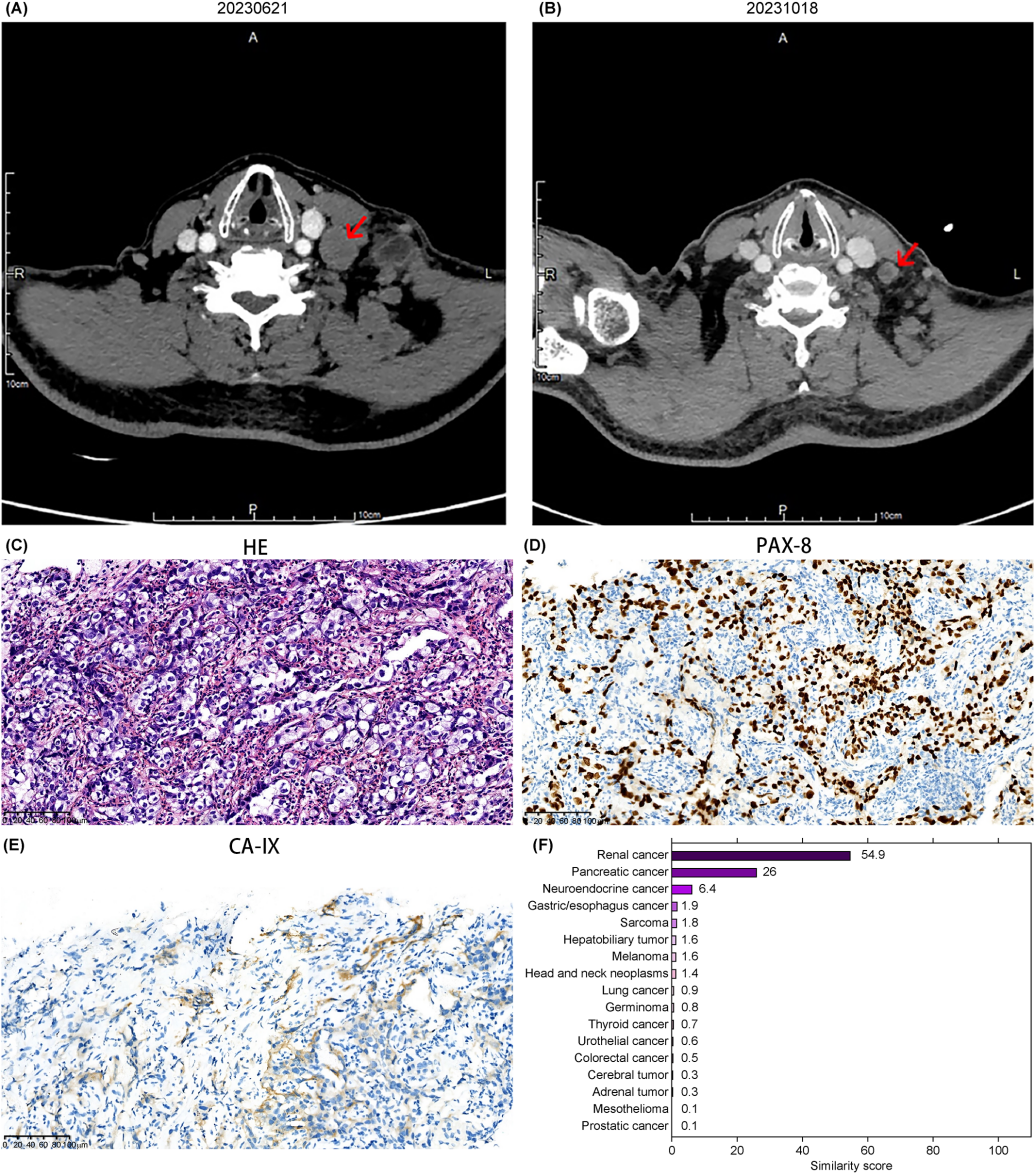
Imaging, pathology, and gene similarity score results of Case 2. **(A)** Neck CT showing an enlarged mass in the cervical region. **(B)** Reduction of the cervical lesion observed on August 9, 2023, following targeted therapy guided by the 90-gene test results. **(C)** HE-stained pathological image of the left cervical lymph node. Immunohistochemical staining for **(D)** PAX-8 and **(E)** CA-IX, respectively (20× magnification). **(F)** Gene similarity score results from the 90-gene test.

A 56-year-old Asian male presented with a left cervical mass the size of an egg for 9 months. He had no significant medical, family, or psychological history but reported a 35-year smoking history (20 cigarettes/day). No prior genetic testing had been performed. Physical examination revealed a firm, smooth, well-demarcated, tender mass approximately 5.0 cm in diameter in the left cervical region. Additionally, a firm, smooth, well-demarcated, non-tender mass approximately 4.0 cm in diameter was palpated in the left axillary region. On June 2023, CT imaging revealed enlarged lymph nodes in the bilateral cervical level II, left cervical levels III–V, and the left supraclavicular fossa, along with slight thickening of the nasopharyngeal posterior wall, suggestive of nasopharyngeal carcinoma with left cervical lymph node metastasis (**Figure 2A**). PET-CT demonstrated increased metabolic activity in the left cervical, supraclavicular, chest wall intermuscular, and axillary regions, as well as in bilateral diaphragmatic crura and the left para-aortic lymph nodes, indicating metastatic involvement. Metabolic activity was elevated in the nasopharynx. Nasopharyngeal carcinoma was considered as a potential diagnosis. On June 13, 2023, nasopharyngoscopy revealed a smooth nasopharyngeal surface with slight thickening of the posterior wall. Nasopharyngeal biopsy indicated active lymphoid hyperplasia, suggestive of reactive proliferation. On June 21, 2023, Epstein-Barr virus DNA testing returned negative. Based on the available findings, the diagnosis of nasopharyngeal carcinoma was not supported, and the condition was classified as a cancer of unknown primary origin.

The 90-gene panel revealed a renal cancer origin with a similarity score of 54.9 (**Figure 2F**). A biopsy of the cervical lesion on July 6, 2023, confirmed metastatic carcinoma in the left lower cervical lymph node, with supplementary immunohistochemical markers suggesting possible metastasis from a high-grade renal epithelial tumor. Immunohistochemistry results wereCD10(-), CAIX(focal+, membranous staining), PAX-8(+), vimentin(+), P504S(+), RCC(-), MUC5AC(-), CA19-9(-), CK19(+), and EBER(-) (**Figures 2C–E**). These findings suggested advanced renal malignancy.

Based on NCCN and CSCO guidelines for kidney cancer, the patient opted for combination targeted therapy with everolimus and lenvatinib, which was well-tolerated throughout the treatment course. A follow-up evaluation on October 17, 2023, showed stable disease with approximately 28% tumor reduction (**Figure 2B**). On April 11, 2024, a chest CT scan revealed a large left pleural effusion and pleural thickening, likely related to tumor metastasis. The PFS time after gene-directed treatment was 9 months. A pleural biopsy was recommended for confirmation, but the patient declined both the biopsy and radiochemotherapy. No additional anti-tumor treatments or efficacy evaluations were pursued thereafter.

### 2.3 Case 3

**Figure 3:**
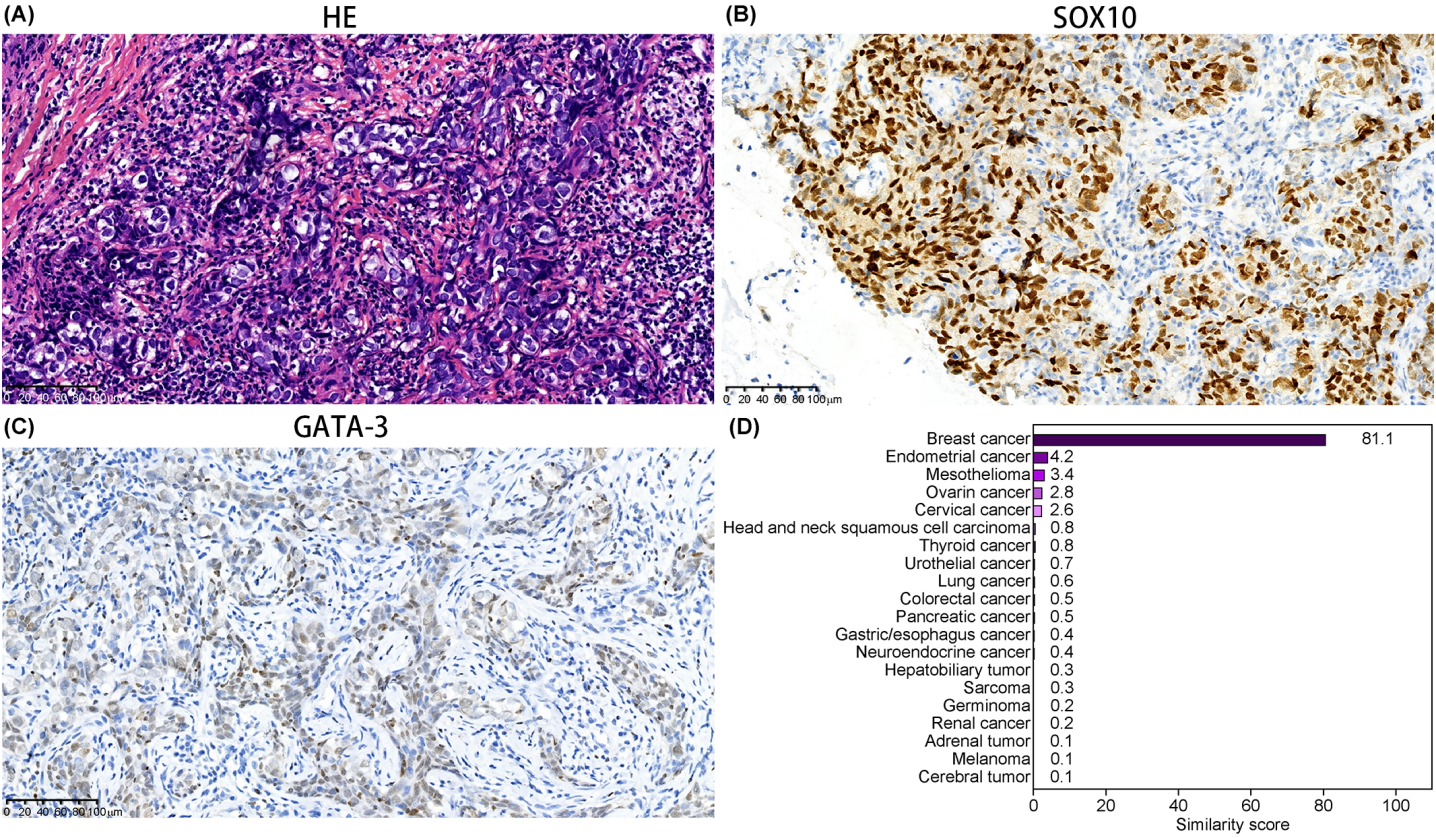
Pathology and gene similarity score results of Case 3. **(A)** HE-stained pathological image of the left axillary lymph node. Immunohistochemical staining for **(B)** SOX10 and **(C)** GATA-3, respectively (20× magnification). **(D)** Gene similarity score results from the 90-gene test.

A 71-year-old Asian female presented with subcutaneous masses in the left cervical region and multiple sites throughout the body for over 20 days. She had no significant medical, family, or psychological history. No prior genetic testing was recorded. Physical examination revealed enlarged lymph nodes in the left axilla, left shoulder, right cervical region, and bilateral inguinal regions. The largest lymph node was located in the left axilla, measuring approximately 2 × 1.5 cm, with clear borders, firm texture, and no tenderness or pain upon palpation. On April 2023, chest and abdominal CT scans revealed enlarged lymph nodes in the left axilla and significantly enhanced nodules in the subcutaneous fat layer of the left upper back and lateral left shoulder. On April 10, 2023, under local anesthesia, the left back mass was excised, and postoperative pathology suggested metastatic squamous cell carcinoma. On April 18, 2023, PET-CT showed increased metabolic activity in multiple nodules in the bilateral cervical level V, left axilla, and left shoulder-back subcutaneous areas, suggesting lymph node metastasis. Nasopharyngeal and esophageal tumors were considered as potential diagnoses. However, further nasopharyngoscopy and gastroscopy did not reveal any tumor-related lesions. Given the presence of multiple lymph node metastases in the neck, back, and axillary regions, which rendered surgical intervention unfeasible, the patient was managed primarily with systemic palliative treatment. Consequently, the patient was diagnosed with CUP and received platinum-based chemotherapy according to clinical guidelines. The first cycle of the PF regimen (5-FU 700 mg days 1–4 + cisplatin 40 mg day 1, 30 mg day 2–3, every 3 weeks) was administered on April 22, 2023, but new enlarged cervical lymph nodes appeared after chemotherapy. A fine-needle biopsy of the left axillary lymph node in May 2023 showed poorly differentiated carcinoma infiltration. Immunohistochemical results: CK-pan (+), P40 (-), P63 (-), PAX-8 (-), TTF-1 (-), GATA-3 (weak +), Ki-67 (+, 60%), SOX10 (focal +), ER (5% cutoff, low positive), PR (-), Her-2 (1+). Molecular pathology results: EBER (-) (**Figures 3A–C**). Current immunohistochemical markers did not support evidence of squamous cell carcinoma, and the primary site could not be determined.

The 90-gene panel indicated a gene similarity score of 81.1 for breast cancer (**Figure 3D**). Despite PET-CT not showing any bilateral breast lesions, the possibility of occult breast cancer could not be ruled out. The patient was ER(5% cutoff, low positive), PR(-), and Her-2(1+). Based on NCCN and CSCO breast cancer guidelines, two cycles of the TC regimen (paclitaxel 210 mg day 1 + carboplatin 300 mg day 1, every three weeks) were administered. The chemotherapy was well-tolerated and resulted in a reduction in the size of the cervical and axillary lymph nodes. The patient did not continue treatment or undergo further efficacy evaluation for personal reasons and passed away in December, 2023 due to disease progression. The PFS time after gene-directed treatment was 7 months.

## 3 Discussion

CUP is defined in the NCCN and European Society for Medical Oncology guidelines as a histologically confirmed metastatic malignant tumor where the primary site cannot be identified through standard diagnostic methods (3, 17). Although CUP accounts for less than 5% of all cancers, its treatment is challenging and prognosis relatively poor. Approximately 80% of patients with CUP show moderate sensitivity to treatment, with median overall survival typically under 1 year. Currently, treatment relies on empirical chemotherapy with platinum-based drugs combined with taxanes or gemcitabine. The efficacy of these regimens has been demonstrated in several trials. Culine et al. conducted a phase II, two-arm, multicenter trial (GEFCAPI 01) evaluating cisplatin with gemcitabine or irinotecan in CUP patients. The cisplatin plus gemcitabine group (GC group, 21 patients) and cisplatin plus irinotecan group (IC group, 15 patients) achieved ORRs of 55% and 38%, respectively; median OS was 8 and 6 months, respectively, showing a higher ORR and longer survival in the GC group (18). Similarly, Greco et al.’s phase II trial assessed docetaxel with cisplatin or carboplatin, showing comparable efficacy (median PFS of 8 months) with similar safety profiles. One-year survival rates were 42% and 29% in the docetaxel plus cisplatin and docetaxel plus carboplatin groups, respectively (19). These studies suggest that platinum-based combinations offer benefits, but the prognosis remains poor, underscoring the need for optimized treatment strategies. In the three cases reported in our study, for the CUP patient suspected to have a breast cancer origin, treatment with a platinum-based doublet regimen (paclitaxel + carboplatin) resulted in a PFS of 7 months. For the CUP with a suspected cervical cancer origin, the treatment regimen involved a platinum-based doublet (with paclitaxel and gemcitabine), achieving an ORR of 25% and a PFS of 9 months. For the CUP suspected to be of renal cancer origin, a dual-target therapy (everolimus + lenvatinib) was used, with an ORR of 28% and a PFS of 9 months. Overall, after identifying potential primary sites and providing targeted treatment, the efficacy slightly exceeded that observed in previous empirical studies.

With advances in molecular diagnostics, various technologies have been developed to improve the accuracy of determining the primary site of CUP, thereby guiding treatment selection and potentially improving prognosis (20, 28). Key molecular techniques include gene expression profiling (GEP), DNA methylation analysis, liquid biopsy-based approaches, and microRNA profiling (21–23). GEP compares tumor gene expression patterns with reference databases to infer the most probable tissue of origin, achieving diagnostic accuracy rates of 82%–97% in multiple studies. Liquid biopsy, primarily through cfDNA analysis, provides mutation and epigenetic information to support treatment decisions. While its accuracy in identifying the primary site may vary, it still offers valuable insights, though GEP remains one of the most widely validated methods. Recently, deep learning models based on cytology images and cfDNA methylation-based classifiers have further improved CUP diagnosis (24). The 90-gene panel used in this study is an optimized GEP-based tool, developed from prior research and validated in multiple clinical trials (25, 26). This method has demonstrated high accuracy in predicting the primary tumor site and guiding personalized treatment selection.

A major strength of this report is the use of a 90-gene panel for molecular diagnosis, which demonstrated high accuracy in identifying the primary site and facilitating tailored treatment strategies. The current case report analyzed three cases of CUP, each originating from a different organ and presenting in varying locations. Treatment was guided by a 90-gene molecular profiling panel and conducted according to existing cancer subtype-specific guidelines. All patients achieved a positive response, with a median PFS of 9 months (range: 7–9 months), notably exceeding the historically reported PFS of 3–6 months in patients receiving empirical chemotherapy regimens (27).

A recent advancement in CUP treatment was seen in the Fudan CUP001 trial (25, 26), researchers used a 90-gene panel based on gene expression profiling to analyze pathological sections and identify the tumor tissue origin with an accuracy rate exceeding 90%, significantly improving diagnostic precision and enabling personalized treatment for patients with CUP. The trial included 182 patients with untreated CUP, with a median follow-up of 42.9 months. Results indicated that the specific treatment group had significantly longer PFS compared to the empirical treatment group (9.6 months vs. 6.6 months, HR = 0.68, 95% CI: 0.49–0.93, P = 0.017), and showed a trend toward longer OS (28.2 months vs. 19.0 months, HR = 0.74, 95% CI: 0.52–1.06, P = 0.098), suggesting a potential therapeutic advantage of molecularly guided treatment in CUP patients.

However, There are still certain limitations existed in the field of CUP detection. First, CUP diagnosis relies on reference databases, and the representation of rare tumor types may be insufficient, potentially leading to misclassification. Second, poorly differentiated tumors or small cell carcinomas may exhibit lower prediction accuracy. Additionally, while GEP typically provides a most probable primary site, some cases may yield multiple possibilities, complicating clinical decision-making. For instance, common cancers (e.g., breast or lung cancer) are well represented in databases, potentially biasing predictions. Future improvements should focus on expanding the reference dataset and refining the algorithm to enhance accuracy.

In this report, we present three cases of CUP with different suspected origins. Based on the 90-gene testing results, individualized treatment plans were implemented, leading to better efficacy compared to previous empirical treatments. This highlights the important role of molecular testing in the diagnosis and treatment of CUP. While molecular profiling has greatly improved CUP diagnosis and treatment, further research is needed to optimize its clinical application. Future studies should validate these approaches in larger cohorts, integrate multiple molecular profiling techniques, and explore multi-omics and AI-driven methods to refine CUP classification, treatment, and predictive accuracy. As precision medicine continues to evolve, incorporating molecular diagnostics into routine clinical practice will likely transform the management of CUP, offering patients more personalized treatment options establishing standardized guidelines for molecularly guided CUP treatment.

## Data Availability

Datasets are available on request: The raw data supporting the conclusions of this article will be made available by the authors, without undue reservation.
